# Supplementary material for: Are carbon emissions trading and green financial instruments synergistic? -Comprehensive quantitative research based on content analysis
Source: PLoS One. 2024 Mar 7;19(3):e0298601. doi: 10.1371/journal.pone.0298601 (PMC10919601; doi:10.1371/journal.pone.0298601)
Supplement: S1 Data — (ZIP) [file pone.0298601.s001.zip › Data/地级市Z_F工作报告.zip!/╕╩╦α/╢¿╬≈╩╨/└┤╫╘╣1⁄2╓┌║┼í╛┬φ┐╦╩2╛▌═°í┐_2018.docx]

各位代表：

　　现在，我代表市人民政府，向大会报告工作，请予审议，并请各位政协委员和其他列席人员提出意见。

一、2017年工作回顾和本届政府开局

　　2017年，是全市经济社会发展极不平凡的一年。面对严峻经济形势和诸多发展困难，市政府在省委、省政府和市委的坚强领导下，深入学习贯彻党的十八大、十九大精神和省第十三次党代会、市第四次党代会精神，坚持稳中求进工作总基调，以脱贫攻坚为统揽，扭住投资不放松，攻坚克难抓落实，全力推动经济社会平稳健康发展。预计全年完成生产总值344亿元，同比增长3%。固定资产投资354亿元，下降43%。规上工业增加值29.5亿元，增长3%。社会消费品零售总额127亿元，增长8%。大口径财政收入47亿元，与上年持平；一般公共预算收入22.7亿元，同口径下降4%；一般公共预算支出210亿元，增长5%。城乡居民人均可支配收入22481元和6918元，分别增长8%和10%。金融机构存贷款余额785亿元和732亿元，分别增长2.6%和9.9%。

　　一年来，我们主要做了以下工作：

（一）聚焦深度贫困，推动脱贫攻坚精准精微。深入贯彻落实中央深度贫困地区脱贫攻坚座谈会精神，以4个深度贫困县、34个深度贫困乡镇和557个深度贫困村为重点，开展精细精确精微的“绣花”式扶贫，预计贫困人口从动态调整后的46.36万人减少到36.24万人，贫困发生率从17.5%下降到14%左右。将最强领导力量、最优帮扶资源向深度贫困县乡村倾斜，市级领导联县包乡抓村，3500多名干部开展驻村帮扶。重点推进产业扶贫，新增市级以上农业产业化龙头企业59家、农民专业合作社1330家、家庭农场158家，带动群众增收致富，农民人均产业收入占可支配收入60%以上，全国产业扶贫现场会在我市成功举办。紧盯基础设施欠账短板，制定出台自然村道路硬化等4个三年实施方案，硬化自然村道路3331公里，实施易地扶贫搬迁8700户3.8万人，建成3万千瓦并网光伏扶贫试点项目，6427户贫困户受益，改造农村D级危房2.25万户，农村自来水普及率达到91.6%，行政村通光纤宽带率达到97%。加快提升公共服务水平，新建“乡村舞台”115个、“一村一场（篮球场）”250个、乡镇体育健身中心10个，覆盖率分别达到100%、90%、80%。新建农村幼儿园184个，农村学前教育毛入学率达到90%；提高建档立卡贫困户住院报销比例，大病兜底政策全面落实。加大脱贫攻坚资金投入，市县区财政用于脱贫攻坚支出174亿元，累计发放精准扶贫专项贷款66亿元、易地扶贫搬迁贷款31.6亿元。福州市在产业发展、劳务输转、项目建设、干部挂职、捐资助学等方面大力帮扶，落实资金1.04亿元，东西部扶贫协作和中央单位定点帮扶工作成效明显。

　　（二）强化措施配套，推动项目建设落地见效。面对投资持续下滑的严峻形势，采取“十个集中”推进措施，集中力量加强项目建设，全力推动投资止滑企稳。实施500万元以上项目1063个、亿元以上项目106个。梯次举行项目开工，连续5次集中开工项目415个，完成投资96亿元，拉动投资增长16.9个百分点。加大协调推进力度，做实做细前期，推行联审联批，优化招标程序，解决推进难题，渭武高速等9个省列重点项目、通渭县中医院医技综合楼等130个中央预算内投资项目、普尔康中药制剂生产线等100个市列重点项目加快建设。认真研究国家最新政策导向，及早谋划论证2018年计划实施项目和“十三五”后三年储备项目，着力补齐光电材料、装备制造、能源环保等十个方面产业短板项目，共谋划储备项目3208个，总投资7163亿元，其中产业短板项目728个，总投资1069亿元。谋划储备PPP项目141个，总投资2097亿元。组团参加兰洽会、敦煌文博会等重大节会，举办福定东西部扶贫协作、福州企业家定西行等招商推介活动，实施招商引资项目317个，总投资350亿元。制定出台固定资产投资正向激励和反向问责办法，项目推进机制不断完善。

　　（三）破除瓶颈制约，推动产业发展提质增效。开展中医药产业发展综合试验区先行先试，推动中药产业、中医事业、中医药学融合发展。成功举办2017甘肃中医药产业博览会，“中国药都”影响力明显提升。启动实施中药产业、中医事业两个“百千万”人才三年培养行动计划，中医药人才缺乏问题开始破题。修订、制定并发布中药材地方标准59项，中药材规范化种植、初加工、仓储、流通标准体系初步形成。组建成立中药材种子种苗产业联盟、规范化种植联盟和科技创新联盟。建立种子种苗繁育基地10万亩，规范化种植基地81万亩。70户中医药企业通过新版GMP认证，陇西一方取得配方颗粒生产资质并批量生产，天士力集团中天金丹药业从金昌分公司划转生产药号14个，已投入生产4个。康美西部中药城建成运营，甘肃中药材交易中心具备上线交易条件。中医药产业总产值达到144亿元，增长8.8%。加快实施马铃薯良种工程和主食化战略，生产原原种10亿粒，建成原种扩繁基地6万亩、一二级种扩繁基地150万亩，主食化加工能力达到12.6万吨。加强与中国环科院技术合作，淀粉加工尾水处理循环利用取得新进展。定西被认定为全国首批区域性马铃薯良种繁育基地和特色农产品优势区，“定西马铃薯”通过国家地理标志产品保护技术审查。马铃薯产业总产值达到163亿元、增长10%。编制完成中国西部草都建设总体规划，谋划总投资103亿元项目40个，种植优质牧草185万亩、加工商品草113万吨，畜禽饲养总量和肉蛋奶总产量分别达到2800多万头只、33万吨，草牧业总产值达到155亿元、增长10.7%。新增经济林果13.3万亩，种植蔬菜81万亩，果蔬产业总产值达到71亿元、增长3%。总投资50亿元的雪榕食用菌产业园建设进展顺利。推进工业供给侧结构性改革，制定“一企一策”行动方案，扶持28户重点企业做大做强，帮助29户停产企业恢复生产。完成重点工业项目投资30亿元，蓝天公司马铃薯复合粉生产线等113个项目开工建设，康华机械智能制造等26个项目建成投产。新增规上工业企业18户、累计达到169户，实现利润7.8亿元、增长120%。现代服务业加快发展，91个文化旅游项目建设进展顺利，文化产业增加值5.7亿元、增长19%，旅游综合收入30亿元、增长28.8%。10个大型物流项目、10个大型农产品批发市场、10个县乡便民市场加快推进，完成投资7.2亿元。陇西、岷县农村商业银行成功改制，陇西神舟村镇银行挂牌运营，金融便民服务点行政村覆盖率达到96%。电子商务和物流快递融合发展，快递企业达到500家，新增网店360个，网上交易额突破10亿元。服务业增加值达187亿元、增长2.6%。

　　（四）着眼增强后劲，推动城乡建设统筹发展。以新型城镇化为引领，加快推进城乡一体化进程，城镇化率达到33.8%、较上年提高1.9个百分点。《定西市城市总体规划（2016-2030年）》获省政府批复，通渭、临洮、渭源县城总体规划启动修编。市区实施城建项目81个、完成投资44亿元，定西北站站前广场等21个项目建成投用，定西体育场等60个项目加快推进，中华路市民公园启动建设，兰州至定西天然气管线全面建成。各县实施城建项目93个，完成投资50亿元，城镇服务承载功能不断提升。 首阳中药材小镇列入国家级特色小镇，3个省级、8个市级特色小镇建设进展顺利。抢抓国家新一轮棚改机遇，制定今后三年6万户棚改攻坚计划，开工建设棚改住房4109套（户）。依托定西“座中联七”区位优势，加快构建“八通十六达”综合交通运输体系。宝兰高铁建成通车，兰渝铁路全线贯通。总投资274亿元的交通PPP项目——通定、定临、陇漳3条地方高速开工建设，岷漳地震灾后重建9条等量置换省道项目启动实施，巉郭公路等8条普通省道建设进展顺利，完成交通项目投资85.6亿元、增长23%。定西支线机场、漳县通用机场、渭河源景区和市医院起降点、凤合高速岷县段、岷漳高速连接线前期工作积极推进。编制完成《引洮水资源高效利用助推精准脱贫规划》，谋划总投资47亿元的六大类项目81个。实施引洮二期供水工程等水利项目59个、完成投资7亿元。实施土地整治项目27.2万亩，新增耕地3.75万亩。

　　（五）坚守发展底线，推动生态环境持续改善。围绕打造国家生态文明先行示范区，争取省发改委批复《渭河源区生态保护与综合治理规划实施方案》，编制总投资103亿元项目308个，开工203个，累计完成投资13.4亿元。实施新一轮退耕还林、三北防护林、天然林资源保护等林业生态重点工程，完成造林封育42.2万亩、面山绿化9.9万亩、生态廊道绿化990公里2.1万亩、义务植树2113万株。实施国家水土保持重点工程等项目47个，新修梯田20.5万亩，新增水土流失综合治理面积350平方公里，治理程度达到55.2%。全面推行“河长制”，建立四级河长名录，落实各级河长巡河制度，启动洮河、渭河、漳河、关川河、牛谷河“管治建”规划编制。统筹推进全域无垃圾城乡环境综合治理“五大工程”，城乡环境整治初见成效。深刻汲取祁连山生态环境问题教训，认真整改中央环保督察反馈问题，23项反馈问题整改销号20项，其余3项完成年度整改任务，123件环境信访事项全部办结。全面排查5个自然保护区生态环境问题，自查发现问题正在整改落实。扎实开展大气、水、土壤污染防治，市区空气质量优良天数比率达85%以上，全市城市饮用水和地下水、地表水监测点水质100%达标，工业集聚区污水实现集中处理，畜禽禁养区划定工作全面完成。

　　（六）注重问题导向，推动改革开放提速深化。农村“三变”改革试点启动实施，“三权分置”改革有序推进，土地确权登记颁证通过省级验收。陇西县建设用地二级市场试点开局良好，农村集体资产股份权能改革全面完成。深入推进“三去一降一补”，扎实开展过剩产能专项整治，房地产去库存185万平方米。深化“放管服”改革，调整19个部门权力事项233项，取消行政审批中介服务事项25项，全市企业信用信息共享监管平台建成运行。“多证合一”改革全面完成，商事制度改革红利不断释放，新增市场主体1.4万户，非公经济增加值占比达到54%。致力破解融资难题，组建成立定投集团。加强融政企对接，投融资渠道有效拓展。营改增等减税政策全面落实，减税9亿元。中药材产值保险在全市推广，承保面积扩大到19.3万亩。全省中药材购销行业增值税进项税额核定扣除试点在我市启动实施。融入“一带一路”对接南向通道，举办发展研讨会启迪思维，凝聚形成“263”基本思路，启动战略研究报告和发展规划编制。坚持引进来和走出去并重，与四川凉山州缔结友好市州，与中国化工、中国建材、北京城建、湖南建工、康美、珍宝岛、阿里巴巴等大型企业签订战略合作协议，与万达、神威、保和堂等知名企业达成合作意向，对外交流合作平台进一步拓展。修订招商引资优惠政策，加强招商项目对接服务，制定推行新型政商关系“双6条”正负面清单，营商环境不断优化。培育外贸进出口主体，新增备案企业11户，渭源县被评为国家级出口食品农产品质量安全示范区。

　　（七）加大投入力度，推动民生福祉不断增进。财政用于民生方面支出134亿元、增长9.8%。省市承诺为民办理实事全部办结。新增城镇就业2.8万人，输转城乡劳动力63.8万人，实现劳务收入125亿元、增长8.7%。探索公办带民办、名园帮民园共建模式，着力解决城区幼儿入园难题；实施义务教育全面改薄项目905个、建设校舍20万平方米，标准化学校建设率达96%，安定、陇西顺利通过国家义务教育均衡发展验收；教育教学质量稳步提升，普通高考二本上线率达34%；加快职业教育特色化发展步伐，甘肃农业大学应用技术学院开工建设。统筹推进医药卫生体制改革，城市公立医院全部实现药品零差率销售，医联体建设、分级诊疗全面实施，县外住院就诊率下降到11.3%。城乡居民基本医疗保险系统率先在全省上线运行，城镇职工医疗保险实现跨省异地就医直接结算。新农合参合率达到98%，住院报销比例达到65%以上。发放城乡低保、特困供养、孤儿生活保障金10.8亿元，城乡医疗救助和临时救助等3.3亿元，特困群体、受灾群众基本生活得到较好保障。《柴生芳》《渭水医魂》等剧目赴省进京演出。漳盐制作技艺等15个项目入选省级第四批非遗保护名录。定西体育馆、国家甘肃临洮体育训练基地建成投用。认定省级科技创新型企业15家，新增高新技术企业7家，选派1100多名科技特派员到企业、贫困村开展服务。安全生产形势总体稳定，食品药品监管不断加强。认真办理群众来信来访，严厉打击各类违法犯罪活动，我市荣获全国社会治安综合治理“长安杯”奖。双拥、民族宗教、外事侨务、地震、粮食、气象、农机以及妇女儿童、青少年、老年人、残疾人、社会慈善等事业都取得新的成绩。

　　（八）健全完善制度，推动自身建设切实加强。认真学习贯彻习近平新时代中国特色社会主义思想，推进“两学一做”学习教育常态化制度化。修订完善市政府工作规则，落实市县区政府法律顾问、公平竞争审查等制度，开展重大行政执法决定法制审核制度试点工作，法治政府建设扎实推进。建立市政府工作周安排月计划季部署制度，协调推进“重中之重”工作160项、“难中之难”工作26项。建立“五步三法”政务督查制度，紧盯不落实的事，追责不落实的人，做到见人见事见效果，督查工作实效性明显增强。深入推进“互联网+政务服务”，市县区网上“晒权、行权、监权”平台建成运行。履行全面从严治党主体责任，加强行政监察和审计监督，开展“三纠三促”“三查三治”纪律作风专项整治行动，扶贫资金使用等重点领域监管工作不断强化。自觉接受市人大及其常委会法律监督和工作监督、市政协民主监督以及社会各界监督，130件人大代表建议、209件政协委员提案全部办结。答复处理电子民生平台诉求近8万件。

　　各位代表！2017年，全市主要经济指标除第一产业增加值、城镇居民人均可支配收入外，其他指标没有完成年初预期目标，特别是投资下滑严重。有项目谋划不足、前期工作不实、重大项目缺乏等原因，有发展基数、统计方式、环保监管等因素，有个别干部不担当、不作为、慢作为等问题。对于这些问题，我们必须高度重视，采取针对性措施，力促经济回稳向好。

　　各位代表！回顾2017年工作，我们清醒地认识到，全市经济社会发展面临的困难和问题依然突出，政府工作面临的压力和挑战依然很多。一是脱贫攻坚任务艰巨，全市还有36万多贫困人口，近70%属贫中之贫、困中之困，越往后难度越大。二是县域经济发展滞后，特色优势产业开发层次低，工业经济总量小，现代服务业和新兴产业培育不足，财政收支矛盾突出，经济发展质量效益不高。三是城乡基础设施欠账大，城镇承载服务功能不强，生态环境脆弱，生态治理和环境保护任重道远。四是对外开放程度低，体制机制不活，科技、人才、资金要素制约明显，新经济、新业态、新模式引进培育不够，共享经济没有破题，经济发展活力动力不足。五是公共服务保障能力弱，关系群众切身利益的就业、上学、医疗、养老、供热供水、安全生产、食品药品安全等方面还存在不少薄弱环节，文化旅游体育发展融合度不高。六是政府职能转变不到位，市场监管有待加强，个别部门服务意识不强，少数干部思想观念保守，创新劲头不足，官本位思想严重，工作落实不力。对此，我们要下决心认真加以解决。

　　各位代表！2017年，面对多年来少有的严峻经济形势，我们直面问题挑战，认真分析研判，打出了抓项目促投资稳增长的组合拳，形成了一些务实管用的制度机制，保持了经济基本面总体平稳，维护了社会大局总体稳定，成绩来之不易。这是省委、省政府和市委坚强领导的结果，是市人大及其常委会和市政协大力支持的结果，更是全市广大干部群众团结奋斗的结果。在此，我代表市人民政府，向全市各行各业的建设者，向各级人大代表、政协委员，向各人民团体、民主党派和无党派人士，向驻定人民解放军、武警官兵和公安民警，向中央和省属在定各单位，向为定西发展作出贡献的老领导、老同志，向所有关心支持定西发展的社会各界人士，表示衷心的感谢和崇高的敬意！

二、2018年重点工作和决胜全面建成小康社会的基础性任务

　　2018年，是贯彻落实党的十九大精神的开局之年，是改革开放40周年，是啃下脱贫攻坚这块硬骨头、决胜全面建成小康社会的重要一年，是实施“十三五”规划承上启下的关键一年。今后三年，是打好脱贫攻坚战、全面建成小康社会的决战决胜期。在重点安排2018年各项工作的同时，通盘考虑今后三年的任务，对实现全面建成小康社会宏伟目标具有决定性、基础性作用。党的十九大绘制了新时代党和国家事业发展宏伟蓝图，作出一系列重大战略决策部署，政策利好加快释放，我国经济长期向好。省委省政府筹集专项资金支持产业发展、人居环境工程改善和基础设施建设，为我们打好脱贫攻坚战带来重大机遇。全市县域经济发展配套政策的出台实施，2018年及“十三五”后三年特别是产业短板项目库的调整充实，工业强市政策措施的配套完善，城市建设“十大工程”的集中谋划，“八通十六达”综合交通运输体系的加快构建，为各项重点工作推进奠定了基础。我们要紧扣我国社会主要矛盾的变化，把握我市经济社会发展的特点，协调推进2018年重点工作和今后三年任务，奋力开创定西发展新局面。

　　政府工作的总体要求是：深入贯彻落实党的十九大精神，以习近平新时代中国特色社会主义思想为指导，全面落实中央经济工作会议精神和省第十三次党代会、市第四次党代会精神，坚持稳中求进工作总基调，以新发展理念为引领，以供给侧结构性改革为主线，紧盯脱贫攻坚，全力打造“中国药都”“中国薯都”“中国西部草都”和全国特色种子种业基地，集中突破项目建设、工业发展、科技人才、对外开放，着力加强城乡建设、生态环保、民生保障、社会治理，积极发展新经济培育新动能，加快发展壮大县域经济，努力推动经济社会平稳健康发展。

　　经济社会发展的主要预期目标是：生产总值增长6%，固定资产投资增长10%，规上工业增加值增长6%，社会消费品零售总额增长8%，大口径财政收入和一般公共预算收入分别增长6%、4%，城乡居民人均可支配收入分别增长8%、10%。城镇登记失业率控制在4%以内。居民消费价格指数控制在3%以内。这些预期指标，是基于当前社会主要矛盾变化和我市经济发展实际提出的，既考虑了发展速度，更注重了发展质量，经过努力是可以完成的。个别指标还需要和省上进一步衔接，如有调整，我们将以市人大常委会审定的为主。

　　（一）坚持精准施策，全力攻坚深度贫困。深入贯彻习近平总书记扶贫开发战略思想，统筹推进区域发展与区域脱贫，紧盯“两不愁、三保障”，集中人力物力财力，瞄准深度贫困聚焦发力，抓实抓细“十大行动”，每年减少贫困人口10万人以上，坚决打好脱贫攻坚战。实施产业扶贫行动，因地制宜发展特色优势产业和多元富民产业，扶持发展贫困人口参与度高的特色种养业、加工业、服务业，拓宽贫困群众增收渠道。培育发展龙头企业、农民专业合作社等新型经营主体，完善与贫困户利益联结机制，提高贫困户进入市场组织化程度，解决贫困群众增收难题。实施技能提升行动，因需施策开展“两后生”培训、农民适用技术培训及技能鉴定，每年精准培训贫困劳动力3万人以上，提高贫困群众自我发展能力。加大定点劳务培训输转力度，提高劳务输转组织化程度，劳动力转移就业人数稳定在60万人以上，实现劳务收入125亿元以上。实施教育扶贫行动，完善就学控辍机制，落实贫困家庭学生教育资助扶持政策，对接实施精准扶贫专项招生计划，确保贫困家庭学生义务教育有保障，阻断贫困代际传递。实施健康扶贫行动，建立健全健康扶贫保障机制，实现大病集中救治覆盖所有深度贫困村，对患大病和慢性病的贫困人口进行分类救治。提高建档立卡贫困人口住院报销比例，减轻群众医疗负担，解决基本医疗保障问题。实施安居扶贫行动，改造农村C级危房1.9万户，2019年完成农村危房改造任务，确保2020年实现贫困群众“住房安全有保障”目标。实施易地扶贫搬迁行动，采取集中安置、插花安置、异地安置等办法，搬迁8691户3.72万人，实现应搬尽搬。完善搬迁后续扶持政策，加快培育发展后续产业，提升搬迁质量和脱贫效果，确保搬迁一户、脱贫一户。实施生态扶贫行动，吸纳有条件的贫困人口参与造林和生态管护，就地增加劳务收入。实施兜底保障行动，推进农村低保制度与扶贫开发政策有效衔接，将符合农村低保条件的建档立卡贫困家庭全部纳入保障范围，实现政策兜底脱贫。实施贫困村整体提升行动，完成自然村道路硬化6000公里以上，2019年自然村全部通硬化路。深度贫困村自来水普及率达到88%，集中供水率达到91%。行政村光纤宽带、动力电实现全覆盖。实施内源扶贫行动，注重扶贫与扶志、扶智相结合，通过生产奖补、劳务补助、以工代赈等方式，将帮扶措施与贫困群众参与挂钩，激发贫困群众内生动力。创办新时代农民脱贫攻坚讲习所，加大政策宣讲和产业培训力度。整合涉农资金、专项资金、社会帮扶资金，创新金融扶贫模式，确保市县区脱贫资金投入稳步增长。深化东西部扶贫协作，重点实施福定工业园、千人培训、生态经济林建设等项目。加强与中央单位定点帮扶对接，深化“千企帮千村”精准扶贫行动，引导社会力量发挥优势开展帮扶。加强贫困人口动态管理，落实行业数据共享比对机制，强化精准识别、精准退出，坚决防止数字脱贫、虚假脱贫。加强扶贫资金和精准扶贫专项贷款使用监管，坚决整治扶贫领域腐败问题，确保扶贫资金安全规范高效运行。

　　（二）坚持投资拉动，全面加强项目建设。牵住固定资产投资这个“牛鼻子”，紧盯国家最新政策导向，结合“十三五”规划中期评估，积极谋划助推脱贫攻坚、补齐产业短板、支撑县域经济、融入南向通道、发展共享经济等方面的大项目好项目。编制完成投资项目“四库五本”，2018年争取实施500万元以上项目800个以上，完成固定资产投资390亿元。积极争取政策性投资项目，力争投资额达到60亿元以上。充实完善PPP项目库，激发民间投资活力，促进民间投资增长10%以上。探索运用“容缺受理”“模拟审批”方式，优化并联审批流程，加快项目前期进度。继续推行项目集中开工，从3月份开始，每半月举行一次集中开工，确保2018年计划实施项目6月底前全部开工建设，确保中央预算内投资项目投资下达后即可开工建设。加快推进高速公路、铁路航空项目建设，通定、定临、陇漳3条地方高速公路完成投资80亿元以上，9月份开工建设总投资412亿元的凤合高速岷县段、岷漳高速连接线、定渭、漳殪4条地方高速公路，年底前开工建设兰州三环、景泰至礼县过境段等8条高速公路，推进广河至临洮高速公路前期工作。扩建定西、临洮、文峰3个高速出口，完成定西北和文峰出口扩建工程。力争定西支线机场完成选址报告批复，岷县通用机场按照支线选址、通航建设标准列入全省规划，漳县通用机场开工建设，建成渭河源景区和市医院起降点。推动陇海铁路定西城区段改线工程及兰州至延安、临夏至定西、兰州至汉中3条境内和过境铁路取得积极进展。

　　（三）坚持富民强县，发展壮大县域经济。实施乡村振兴战略，落实县域经济发展配套政策，筹集产业发展基金3-5亿元，撬动各类资金30亿元以上，做大做强特色主导产业，夯实县域经济发展基础。

　　支持县区发展壮大特色主导产业。安定区重点发展马铃薯、草牧和现代物流业，打造全国马铃薯产业“洼地”、西部最大的草牧“基地”和面向全省服务全市的现代服务业“平地”。通渭县重点养好畜、发好电、写好字、用好水、种好树（草），一次性导入花椒、金银花等优势特色种植业产业化模式，创建全国现代旱作循环农业示范、新能源精准扶贫示范、“书画+”农耕文化休闲旅游“三大基地”。陇西县重点发展中医药、现代物流和文化旅游产业，抢占中医药绿色发展新高点， 打造李氏宗亲寻根祭祖新圣地，建设“一带一路”现代物流新格局。临洮县重点发展现代农业、现代物流和旅游体验产业，加快建设洮河绿色经济带，主动融入兰州都市经济圈。渭源县围绕“南薯北药、薯药强县、旅游富民”，重点发展马铃薯种薯、中药材和生态旅游产业，打造中医药产业基地、马铃薯种薯繁育基地和大健康生态旅游富民基地。漳县围绕保护开发“两山一井”，加快发展旅游体验和盐岩产业。岷县围绕“一业（当归）为主、多种经营、南来北往”，加快发展中医药和现代物流业。经过三年努力，各县区经济总量在全省排名位次有较大前移。

　　推动特色优势产业转型升级。中医药产业坚持“以医带药、以药促医、医药并举、药医共荣”，打造从GAP种植到GCP应用的全过程标准化生产链、医药大健康产业链和中医药学文化产业链。推广中药材工厂化育苗技术，建立集约化育苗基地500亩，建设规范化种子种苗繁育基地8万亩、标准化种植基地120万亩。积极对接康美、神威、珍宝岛、奥凯等企业，加快标准化种植基地、精深加工和智慧药房建设。推动甘肃中药材交易中心规范运行，提高甘肃中药材价格话语权。加快中医药博览会永久性场馆建设，打造集体验性、生态性、支撑性、国际性、社会性于一体的中医药学综合体，力争8月份部分建成投用。马铃薯产业以鲜品化、主食化、工业化为主攻方向，原原种生产稳定在10亿粒以上（主食专用品种5亿粒），建设百万亩优质种薯扩繁基地。通过三年努力，原原种生产能力占全国一半以上，原种、一级种生产能力占全省一半以上，打造全国最大的脱毒种薯基地。支持巨鹏、薯香园、清吉、田地等主食化加工企业开拓市场，打造全国有影响力的主食化加工基地。提高国家级定西马铃薯交易中心运营水平，加快二期建设进度。实施定西马铃薯系列产品进高校计划，构建“线上线下融合、农超农校对接”营销体系。草牧产业坚持“立草为业、草畜并举、生态优先、绿色发展”，建成优质苜蓿、红豆草、燕麦、岷山猫尾草、岷山红山叶等牧草良种繁育基地6万亩，新种优质牧草180万亩。扶持民祥、天耀、巨盆、现代、方正等加工企业发展壮大，重点发展裹包全贮、青干草等优质草产品，加工商品草150万吨。新建规模养殖场100个、家庭养殖场1000个。扶持中天羊业、西泰等肉类加工企业发展壮大，肉产品加工突破2万吨。在安定、陇西建设大型活畜交易市场，带动草牧业快速发展。种子种业完成发展规划编制，通过引进国内外大型种业公司，建设以道地中药材种子种苗、马铃薯种薯、高原夏菜、特色牧草、优良畜种、苗木花卉为主的特色优势种子种业基地，力争用三至五年时间将种子种业发展成为与中医药、马铃薯、草牧并驾齐驱的第四大产业。果蔬产业要打响高原夏菜品牌，加强冷链物流配套，扩大引洮灌区沿线高原夏菜、设施蔬菜种植规模，种植面积稳定在80万亩以上。因地制宜发展苹果、核桃、油用牡丹等区域性产业，新增林果基地10万亩。

　　培育壮大战略性新兴产业。生物制药重点支持金羚药业、聚信生物等骨干企业与中国农科院兰州牧药所等院校合作，加大精品肝素钠原料药生产支持力度，加快规模化高效分离与制备技术、中药新剂型技术等的应用与推广。现代物流业重点加快岷县多式联运中心、陆港中心、保税物流中心和临洮中铺、安定、陇西现代物流港建设，发展通道经济，打造“一带一路”及南向通道重要物流节点。编制全市市场建设和市区商业网点建设规划，实施渭水源等5个中药材市场标准化改造提升项目，建设甘肃南部农产品等10个批发市场。文化旅游业重点抓好华盛影视旅游文化创意园等33个文化产业项目，开工建设通渭书画文化产业孵化基地、马家窑文化展示传承保护中心等项目。谋划建设以文化创意、文化传播为方向的文化项目，大力挖掘定西文化内涵，加大宣传推介力度，向世界展示文化定西。文化产业增加值增长18%。编制实施全域旅游发展规划，推进临洮、漳县全域旅游示范县创建，建成运营“智慧旅游”平台，完善渭河源、贵清山/遮阳山等景区配套设施，打造一批文化寻根、黄土风情、中医康养、避暑纳凉等全域旅游系列产品。加强与渭河沿线城市、“丝绸之路旅游带”合作，主动融入南向通道旅游圈。开展以宾馆、餐饮、接待服务为内容的技术比武比赛，提升旅游服务整体水平。旅游接待人数和综合收入增长25%以上。新能源产业重点抓好通渭风电基地接入系统设计评审，争取尽快审查批复，建成并网20万千瓦风电项目。7县区全面启动村级光伏电站建设。加快推进陇西城镇生活垃圾焚烧发电等项目。

　　（四）坚持工业强市，推动工业转型升级。我市经济发展最薄弱的环节是工业经济，发展工业经济最重要的是加快工业企业发展。要落实推进工业强市战略配套政策措施，一手抓老企业和传统产业改造提升，一手抓新兴产业培育和招商引资。加快重点工业项目建设，新建、续建陇西一方现代中药自动化提取等千万元以上工业项目89个，完成投资48亿元，工业固定资产投资增长10%以上。理顺经济开发区和工业集中区管理体制，完善基础配套、功能定位及产业布局，定西经济开发区作为市级独立经济单元，重点发展创新型制造产业，各县区经济开发区和工业园区作为县区级独立经济单元，重点发展特色型配套产业，建设发展指标要单独下达，新入驻企业要达到50家以上。引进战略投资者，采取兼并重组、股权投资等方式，促进中医药、马铃薯、牧草精深加工、有色冶金、新型建材、装备制造等工业主导产业做大做强。鼓励支持企业抓技改、创品牌、做龙头，实施顺泰铝业10万吨建筑型材等技改项目82个，完成投资26亿元。落实扶持企业发展政策措施，实施中小微企业培育行动计划，新增中小微企业100户以上、规上工业企业15户以上；支持中小企业创新发展，推动企业与高校、科研院校联合设立研发机构和技术转移机构，加强新技术新产品研发及孵化应用，提高企业市场竞争力；启动中小企业发展基金和企业转贷周转金，多措并举解决企业融资难题，支持凯凯农科、高强度紧固件等企业挂牌上市；支持金融机构优化信贷结构，创新产品服务，突出主业、服务实体，全力支持中小企业发展。工业经济本质上是工业企业经济，工业企业经济本质上是企业家经济。要弘扬企业家精神，提高企业家地位，保护企业家财富，激发企业家活力，发挥企业家作用，促进企业家健康成长。

　　（五）坚持城镇带动，统筹推进城乡建设。主动融入兰（州）西（宁）城市群和关（中）天（水）经济圈，合理规划布局城乡基础设施建设，加快城乡一体化进程。从2018年起，市区及各县连续三年实施“十大城建工程”，力争城市建设一年大改观、三年大变化。年内新建、续建城建项目200个，完成投资120亿元以上。市区编制完成“海绵城市”“智慧城市”“城市双修”等专项规划。围绕提升通畅水平，实施定临路西川段、南环路中段快速通道等道路改造工程，打通中华路南段、正龙路东段等4条“丁字路”，建设东河、气象等6座桥梁，构建“南北通、东西畅”的交通路网。围绕完善服务功能，加快编制城市天然气应用规划，实施天然气管线建设工程，完成民主路、公园路等重点路段管网铺设。科学规划建设公共服务设施，中华路市民公园完成地下工程及广场铺装，定西体育场完成主体工程建设，城市展览馆完成布展投入使用，启动实施西川便民市场、新城区智慧农贸市场、交通路停车场等项目，建成品质便民夜市1处，万达城市综合体落地建设。多渠道筹措资金，全面排查解决城市供热、供水、供电及小区物业管理突出问题。整合公共交通资源，优化市区公交线路，方便群众快捷出行。围绕优化城市生态，实施东河、西河、关川河“三河六岸”景观绿化工程，打造“三河”交汇景观平台。实施东湖公园、薯都大道、永定西路等绿化改造提升工程。实施大碱沟水景观工程和小流域综合治理项目。围绕优化教育布局，盘活中和教育港资源，设立新城区独立高中，新建新城区公办幼儿园1所，完成建成小区幼儿园补建工程，激发新城区发展活力。坚持“双提高、双完善”，全市实施棚户区改造2.5万户以上，建设城市综合体13个。加快培育建设特色小镇，新规划建设市级特色小镇12个，争取元古堆扶贫创业旅游小镇、榜罗红色旅游小镇创建为国家级、省级特色小镇。推动“厕所革命”由景区向全域、由城市向农村、由封闭向开放延伸。实施虎龙口至北顺公路等11条普通省道项目，完成投资6亿元以上。实施定西汽车站搬迁、定西综合客运枢纽建设项目，完成投资5500万元以上。加快引洮二期配套、农田水利等工程建设，完成投资8亿元。

　　（六）坚持生态立市，加强生态环境治理。实现定西永续发展，必须坚持生态立市。牢固树立“绿水青山就是金山银山”理念，落实主体功能区规划，加快生态保护红线、永久基本农田、城镇开发边界三条控制线划定工作，统筹推进山水林田湖草系统治理。组织实施新一轮退耕还林、三北防护林、天然林保护等林业生态重点工程，完成造林封育18万亩，城镇面山和生态廊道绿化8万亩，全民义务植树和农村“四旁”绿化2000万株，森林覆盖率提高到12.2%。实施渭河源区生态保护与综合治理规划，加大项目资金争取力度，引入PPP、EPC等模式，拓宽项目融资渠道，续建、新开工项目130个，完成投资15亿元。争取实施渭河源规模化林业总场建设试点。坚持“三分造、七分管”，建立相邻县区间协调统一、共建共管、联防联治机制，严厉打击破坏林地、森林、草原等自然资源的行为。实施国家水土保持重点工程、坡耕地水土流失综合治理、土地整治、淤地坝除险加固、地质灾害防治等项目，治理水土流失面积350平方公里。实施引洮水高效利用“六大工程”，推进安定、陇西、临洮、通渭城区面山通道生态供水工程建设。建成“河长制”智慧平台，落实各级河长责任，加强河流综合治理。经过三年努力，把市级5条河流打造成城市生态线、发展生命线、群众生活线。开展安定和陇西城区生活垃圾强制分类试点，提高城市生活垃圾无害化处理水平。深入开展全域无垃圾城乡环境综合治理，加大沿路、沿河、沿川及城乡接合部环境整治和陈年垃圾清理力度，逐步建立垃圾收集、转运、处理体系。实行最严格的生态环境保护制度，扎实推进5个自然保护区和林业生态问题整改。坚决打好污染防治攻坚战，加大燃煤锅炉整治、煤质管控和扬尘管理力度，改善城区空气质量。加快城镇生活污水处理设施建设，经济开发区、工业集中区废水全部实现集中处理和在线监控。落实土壤污染综合防治措施，提高农业面源污染治理水平。

　　（七）坚持创新驱动，强化科技人才支撑。没有什么要素比科技要素更具活力，没有什么人才比科技人才更有潜力，没有什么动力比创新驱动更为强劲。深入实施创新驱动战略，深化科技体制改革，科技进步贡献率达到48%。启动建设定西现代科技园，规划建设陇西、临洮、岷县3个经济开发区和安定、通渭、渭源、漳县4个工业集中区科技企业孵化器。强化企业创新主体地位，依托省级重点实验室、工程技术研究中心，加强重点领域关键技术突破和集成应用，引进开发新产品、新技术10项，新增高新技术企业2家、省级科技创新型企业5家，成立陇药产业技术创新战略联盟。争取奥凯种子机械公司院士工作站在定西挂牌，定期召开专家联盟年会。实施科技成果转移转化“双十”计划，建成科技成果转移转化示范机构3个、示范企业10家。推进国家知识产权试点市和安定区国家知识产权强县试点建设，培育知识产权优势企业3家，申请专利850件。鼓励市内科技人员以技术等生产要素作价出资，创办科技型企业，让科技人员在创新活动中得到合理回报、获得应有荣誉。加强和改进科技特派员工作，打造全省科技特派员示范市。注重本土人才培养使用，实施中药产业、中医事业人才培养三年行动计划，引进高层次紧缺人才和专家团队，培养千名应用技术型人才、中医康养人才和省市名中医、医务骨干，培训万名中药产业、中医事业适用人才。实施东西部扶贫协作千人培训计划，三年培训提升金融、资本、网络、企业管理人才1000名。实施工业经济、特色产业、数据信息、教育文化等领域高端人才引进、紧缺型人才培育计划，培养工业企业人才3000名、文化艺术人才100名、教学名师和“双师型”骨干教师370名，办好百名校长和百名教师培训班。鼓励引导高校毕业生向企业流动，支持企业引进高端人才，缓解企业人才短缺问题。健全高层次紧缺人才引进机制，树立“不求所有、但求所用”的柔性用人理念，建立人才培养资金、政策、平台等保障机制，营造良好的人才发展环境。

　　（八）坚持共享发展，培育新经济新动能。以新产业为支撑、以新业态为引擎、以新模式为突破，构建数字经济、网络经济、共享经济、物流快递等新经济产业体系。实施“互联网+智能制造”行动，加快工业化与信息化深度融合发展，在重点领域推进网络化研发、智能化生产、个性化定制、协同化制造，拓展网络信息技术在设计、生产、运营等核心环节的深入应用，探索以开放共享为特征的产业发展新模式。实施“互联网+现代农业”行动，利用互联网提升农业生产、经营、管理和服务水平，发展信息化农业、创意农业、工厂化农业。依托临洮国家现代农业产业园、安定国家现代农业示范区、陇西农村一二三产融合发展试点示范区建设，实施一批集循环农业、创意农业、农事体验于一体的田园综合体项目，示范带动农业新业态发展。推进“互联网+流通”行动，加快安定、岷县、渭源国家电子商务进农村示范县建设，对接阿里巴巴等大型电商交易平台，促进线上线下服务特色产业快速发展。探索“互联网+文化”等经营模式，搭建定西特色文化创意产品线上平台，推动马家窑文化、李氏文化、通渭书画、洮砚文化等优秀传统文化传播，培育发展文化创意设计产业。实施“旅游+”融合工程，促进旅游与文化、体育、教育、中医药、健康养老等互动融合发展。发挥定西道地中药材优势，培育建设一批医养游特色小镇，培训培养一批针灸理疗专业人才，积极发展中医康养产业。创新与融资租赁、创投基金、资产管理公司合作模式，探索发展互联网金融。强化新经济规划引领，研究建立支持新经济发展的体制机制，促进人才、技术、资金、创意、信息等要素向新经济领域流动。落实全省促进大数据发展三年行动计划，实施4G、5G网络建设项目，加快“定西云”建设，推进互联网、物联网、大数据、云计算等技术广泛应用。鼓励扶持“双创”“众创”“众筹”“众扶”等新型创业，为新经济发展提供内生动力。

　　（九）坚持改革开放，有效激发动力活力。深化供给侧结构性改革，落实去产能各项任务，多措并举降低企业成本，加快建立多主体供给、多渠道保障、租购并举的住房制度。全面推开农村“三变”改革，加快集体资产清产核资和确权登记，引导市内外龙头企业、农民专业合作社等新型经营主体积极参与，做到一切皆变、能变就变、变就变好，导入农村发展新要素，激发农村发展新动能。创新互助资金使用模式，增加贫困群众股权收入，壮大村级集体经济。深化投融资体制改革，完善融政企对接机制，大力发展直接融资。依托定投集团，通过发行企业债券、培育发展规范运营各类投资基金和产业基金，增强服务实体经济的能力。编制完成融入“一带一路”对接南向通道发展规划，谋划实施南向通道建设项目。高标准承办2018甘肃中医药产业博览会等节会，筹办“一带一路”中药材采购大会、全国中医院院长大会和医疗器械展销大会，举办定商外商籍才大会，办好马铃薯美食节、药膳（食）节、中医康养节，加快发展会展经济。开展“双招双引、对标福州”活动，坚持招商招资与引智引力并重并举，形成多层次招商引资、全方位驱动发展良好格局，招商引资到位资金增长10%以上。鼓励支持市内企业“走出去”开拓市场，扩大特色优势产品出口份额，外贸进出口总额增长8%以上。

　　（十）坚持以人为本，切实保障改善民生。把就业作为最大民生，落实就业创业政策，新增城镇就业1.7万人以上。坚持优先发展教育事业，启动实施教育振兴三年行动计划，学前教育抓普及、义务教育促均衡、高中教育提质量、职业教育创特色，优化学校结构布局，合理配置教育资源，全面提高教育质量，打造教育强市。科学规划建设城区及较大乡镇幼儿园，严格落实住宅小区配建移交办园规定，缓解城区公办幼儿园入园压力。实施全面改薄项目500个，确保通渭、渭源义务教育均衡发展通过国家验收。加强省级示范性高中建设，支持定西一中、陇西一中、临洮中学打造省级名校。完善定西职业教育集团运行机制，促进县区“一校一特”职业教育特色发展，建成甘肃农业大学应用技术学院一期工程。按照“一区两校”模式，协调定西师专与甘肃中医药大学逐步剥离、实现独立办学。按照“一校两制”模式，争取在定西工贸中专建设定西职业技术学院。完善分级诊疗制度，加快健康信息平台建设步伐，增加群众医改获得感。积极争取甘肃中医药大学附属医院在我市设立分院，加强市县乡村四级中医科室、中医馆建设，推动中医事业快速发展。实施贫困村文化服务提档升级工程，开展“三下乡”“百姓文化广场”等文化惠民活动。市县公共体育场、体育馆覆盖率分别达到100%、88%。推行归口化管理、市场化运作模式，提高定西大剧院、定西体育馆等公共服务设施运营水平。加快文化旅游体育融合发展，坚持品质化原则，做到每季有大型比赛、每月有文化展演、每周有群众活动。全面放开养老服务市场，多形式发展养老服务业。加快市县区残疾人康复和托养中心建设，提升残疾人保障和服务水平。落实粮食安全责任制，提高区域粮食安全保障水平。

　　（十一）坚持服务为先，加强创新社会治理。落实“谁执法谁普法”责任制，深入开展“七五”普法宣传教育。做好社会稳定风险评估，加强社会矛盾纠纷风险预警和排查化解。落实综治领导责任制，加强各级综治中心建设，完善立体化社会治安防控体系。集中整治社会治安突出问题，严厉打击严重暴力犯罪和非法集资、电信网络诈骗等涉众型经济犯罪，打击“两抢一盗”等多发性侵财类犯罪，加强公共安全管理，提高人民群众的安全感和满意度。加强和完善城乡社区治理，持续推进网格化管理、信息化建设、精细化服务，推动社会治理重心向基层下移。落实信访工作责任制，规范信访工作程序，推进依法分类处理信访诉求，执行领导干部定期接访下访和包案督办制度，解决群众合理诉求，提升信访工作法治化水平。深化安全生产领域改革发展，严格安全生产责任制，强化企业主体责任、属地管理责任、部门监管责任，加大源头管控，加强农村生产安全和通道安全，坚决遏制较大以上事故发生。坚持以防为主、防抗救相结合，提高防灾减灾救灾能力。全面贯彻党的民族宗教政策，促进民族团结、宗教和顺、社会和谐。加强国防动员和“双拥”工作，支持工会、共青团、妇联、科协、残联等群团组织工作。加强新闻出版、广播影视、气象、外事侨务、人民防空、老龄、地方志、档案、红十字会、慈善等工作。

　　（十二）坚持依法监管，营造公平市场环境。加大城市规划执法力度，查纠违法占地、违规建设以及不履行合同义务、不配建服务设施行为，维护规划的严肃性和权威性。落实国有建设用地批后监管动态巡查制度，加大城市批而未供和闲置土地处置力度，提高土地利用率。全面完成市公共资源交易平台电子化建设，强化对各方交易主体的监管，规范中介机构和评标人员从业行为。坚持扶优扶强与打假防伪相结合，加大驰名商标和知名品牌保护力度，依法打击制售假冒伪劣商品行为。强化食品药品、农业投入品等的监管，确保食品药品和农产品质量安全。强化计量器具、特种设备监管，重视民生领域和重点行业价格监督，规范电商、微商等新消费领域经营行为，保障消费者权益不受侵害。落实公平竞争审查制度，释放市场主体活力，催生经济发展新动能。坚持依法监管和行业自律相结合，促进旅游、酒店、餐饮等行业规范品质经营。加强金融监管，完善风险应急处置机制，隔离政府债务风险和市场风险，有效维护金融安全。推进质量诚信体系建设，落实“红黑榜”制度，倒逼企业履行质量安全主体责任。加大失信惩戒力度，对失信主体在工程招投标、土地拍卖利用、政府采购、银行信贷等方面实行联合惩戒，让失信者“一处失信、处处受限”。健全预防和解决拖欠农民工工资问题长效机制，切实保障农民工劳动报酬权益。深化商事制度改革，大力推行“双随机一公开”监管，营造公开透明、平等竞争的市场环境。

　　各位代表！以上十二个方面，既是2018年重点工作，又是今后三年决战脱贫攻坚、决胜全面建成小康社会的基础性任务。其中脱贫攻坚是一号工程、重中之重和底线性任务，其他十一个方面都是围绕脱贫攻坚来谋划、设计和安排的，是脱贫攻坚的支撑和保障。市政府将统筹兼顾、履职尽责，以脱贫攻坚为统揽，以“两不愁、三保障”为底线性要求，既不降低标准，也不吊高胃口，合理安排时序，注重质量脱贫，不搞扎堆脱贫，集中有限时间、集中有限力量、集中解决有限的问题，以区域发展推动区域脱贫，以区域脱贫促进一家一户和每个人的脱贫。

三、坚持科学管理建设人民满意政府

　　政府是人民的政府，必须始终坚持为人民服务。只有坚持科学管理，才能更好地为人民服务。只有把人民的事情办好，才是人民满意的政府。我们要以改革创新精神，加强和改进政府管理，努力建设法治高效、勤政廉洁政府。

　　（一）必须科学决策。建立经济社会发展咨询专家库和重大决策咨询论证机制，健全重大事项技术咨询、决策评估、公示听证制度，开展经常性地调查研究，广泛听取民主党派、人民团体、广大群众和社会各界的意见建议，完善落实公众参与、专家论证、风险评估、合法性审查和集体讨论决定等法定程序，提高决策的科学化、民主化、法治化水平。坚持重大事项向市委请示报告制度，严格执行市人大及其常委会决议决定，自觉接受人大法律监督、工作监督和政协民主监督。

　　（二）必须依法行政。落实法治政府建设实施纲要，坚持依法行政一把手责任制，把政府工作全面纳入法治轨道。坚持政府学法常态化，全面落实政府法律顾问制度。强化各级政府和公务员队伍法治教育，提高法治意识和依法行政能力。试点推进行政执法公示、全过程记录和重大执法决定合法审核等制度，做到严格规范、公正文明执法。发挥审计监督作用，强化公共资金、国有资产和领导干部履行经济责任情况审计，全面开展领导干部自然资源资产离任审计。

　　（三）必须从严理事。严格执行市政府工作规则，全面正确履行政府职能，解决好经济职能和社会职能不协调问题。履行经济调节职能，以项目建设为抓手，调整优化经济结构，加快转变发展方式，提高发展质量效益，保持经济平稳健康发展。履行市场监管职能，推进公平准入，规范市场执法，依法对市场主体及其行为进行监督和管理，维护公平竞争的市场秩序。履行社会管理职能，依法管理和规范社会组织、社会事务，化解社会矛盾，维护社会公正、社会秩序和社会稳定。履行公共服务职能，着力促进教育、卫生、文化等社会事业健康发展，推进基本公共服务均等化。

　　（四）必须效能第一。深化“放管服”改革，持续简政放权，争取市级行政许可事项精简30%以上。推行并联审批制度改革，加快推进审批服务事项标准化工作，实现政府投资项目审批6个月内办结。建立一个印章管审批服务模式，畅通行政审批服务渠道。深入推进“互联网+政务服务”，构建“大平台”、融通“大数据”、整合“大系统”，打破信息孤岛和数据壁垒，实现信息资源共享。树牢“最多跑一次”服务理念，建立24小时便民服务平台，完善电子民生、“两微一端”等互动平台，推动实体政务大厅与网上办事大厅深度融合，实现政务服务“一网通办”。

　　（五）必须百姓至上。始终把百姓的痛点、难点和堵点作为政府工作的重点，紧盯百姓普遍关心的柴米油盐酱醋茶、水电路气房学医等民生问题，一件一件地解决、一项一项地落实。加大政务公开力度，落实政府新闻发言人制度，主动回应社会关切。认真办理人大代表议案建议和政协委员提案，开展政风行风民主评议，畅通群众来信来访、建议征集、市长热线等渠道，及时解决百姓反映的困难和问题，不断增强百姓的幸福感和获得感。

　　（六）必须廉洁从政。严格履行政府党组全面从严治党主体责任，认真落实“一岗双责”，深入推进政府系统党风廉政建设。认真贯彻党内政治生活若干准则、党内监督条例、党组工作条例等党内法规，严守政治纪律和政治规矩。严格执行中央八项规定实施细则和省、市相关规定，坚决整治“四风”突出问题特别是形式主义、官僚主义新表现。规范行政权力运行，在公共资金使用、国有资产运营、工程招投标、土地出让、行政审批等重点领域，建立严格的监督制度和权力制约机制，让权力在阳光下运行。

四、下决心转变改进工作作风推动党的十九大精神在定西大地落地生根

　　“一分部署、九分落实”。当下群众反映最多的是我们的工作作风问题，最不满意的是我们的工作落实问题。我们将坚持问题导向，下决心转变改进工作作风，着力解决能力不足不会为、动力不足不愿为、担当不足不敢为等问题，以作风好转促进各项工作落地见效，以工作落实推动党的十九大精神在定西大地落地生根。

　　（一）下决心解决能力不足“不会为”的问题。针对部分干部知识欠缺、学习转化能力不足、思想观念保守等问题，加大培训教育力度，加快补齐网络、金融、资本、企业管理等知识短板，提高适应新时代、贯彻新理念、运用新技术、推动新发展的能力和水平。强化政策学习研究，找准国家政策和定西实际的契合点，及时把政策转化为发展项目。教育引导各级干部大力解放思想，培育世界眼光、战略思维的格局，历练开放包容、主动融入的胸襟，强化开拓创新、不甘落后的意识，弘扬“三苦”精神、发扬“马上就办、真抓实干”作风，以观念的大转变、思想的大解放、知识的大更新、能力的大提升和作风的大改进推动定西大发展。

　　（二）下决心破除动力不足“不愿为”的顽疾。针对部分干部安于现状、落实不力等问题，深入开展“不忘初心、牢记使命”主题教育，学习贯彻党的十九大精神和习近平新时代中国特色社会主义思想，树牢“四个意识”，提高政治站位，增强加快发展的使命感和责任感。弘扬忠诚老实、公道正派、实事求是、清正廉洁的价值观，整治庸、懒、散、慢、拖、装、推、扯、假、滑等庸政懒政怠政行为，营造干事创业良好氛围。“指导千遍不如督查一遍，动员千次不如问责一次”。加大督查问责力度，严格落实“五步三法”政务督查制度，紧盯不落实的事情，跟踪督查不落实的单位，严肃约谈问责不落实的干部，推动督查、约谈、问责制度化、常态化。

　　（三）下决心走出担当不足“不敢为”的困局。针对部分干部不想担责、不愿担责、不敢担责、“生怕树叶掉下来砸破头”的思想倾向，坚持“三个区分”，落实容错纠错机制，让履职尽责者轻装上阵、让敢于担当者消除顾虑、让改革创新者砥砺前行，激励各级各部门紧握奋进之笔、书写得意之作，每年谋划准备三五件打基础、有影响、利长远的事情，努力干成三五件啃骨头、有创新、能过瘾的事情。加强和改进政府绩效管理，突出脱贫攻坚，细化工作任务，明确责任分工，完善考核机制，强化结果运用，不能形成干的不如站的、站的不如看的、看的不如说风凉话的坏风气，不能形成干多干少一个样、干好干坏一个样、干与不干一个样的怪现象。

　　各位代表！打好脱贫攻坚战留给我们的时间只有一千多天。时不我待，我们要只争朝夕。实现各项目标任务，关键是要“干”字当头，埋头苦干、团结巧干、撸起袖子加油干、结合实际创造性地干，咬定青山不放松，不达目的不罢休。越是处于困难期，越是爬坡过坎时，越要脚踏实地干，越要久久为功干，雷厉风行干，精益求精干，以实干推动发展，以实干赢得未来！

　　各位代表！新时代要有新气象，新征程要有新作为，新情况要有新办法。让我们更加紧密地团结在以习近平同志为核心的党中央周围，在习近平新时代中国特色社会主义思想指引下，在省委、省政府和市委的坚强领导下，凝心聚力、开拓创新、真抓实干，为打好脱贫攻坚战、全面建成小康社会、加快建设幸福美好新定西而努力奋斗！
